# Supplementary material for: Submarine slope failures due to pipe structure formation
Source: Nat Commun. 2018 Feb 19;9:715. doi: 10.1038/s41467-018-03176-1 (PMC5818647; doi:10.1038/s41467-018-03176-1)
Supplement: Supplementary file 1 — Supplementary Information [file 41467_2018_3176_MOESM1_ESM.pdf]

Submarine slope failures due to pipe structure formation

Elger et al.

Supplementary Note 1

The indexes (1 to 14) of the coinciding evidence for hydrates and landslides in Fig. 1 refer to the studies of Dobson et al. in the Gulf of Alaska [1], Scholz et al. offshore Vancouver Island [2], Paull et al. offshore the southeastern United States [3], Leslie et al. on the Colombian margin [4], Krastel et al. off northern Argentina and Uruguay [5], Ben-Avraham et al. off South Africa [6], Sultan et al. offshore Nigeria [7], Li et al. offshore Mauretania [8], Bugge et al. on the Storegga Slide [9], Elger et al. on the Fram Slide Complex [10], Dewangan et al. in the Bay of Bengal [11], Horozal et al. in the Ulleung Basin [12], Bangs et al. offshore southwestern Japan [13] and Mountjoy et al. on the Hikurangi Maring [14].

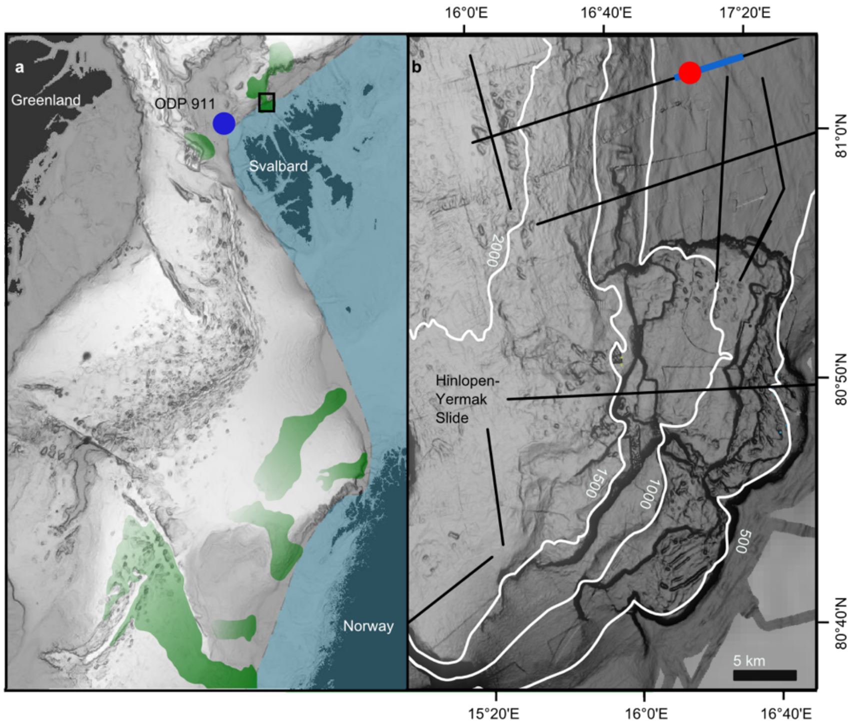

Supplementary Figure 1 | Location of the study area on the continental margin off Svalbard. (a) Overview map with the maximum ice extent since 100 ka (blue area) see [16], location of ODP Site 911 (blue dot) and the areas affected by the Storegga, Sklinnadjupet, Trænadjupet, Andøya, Bjørnøyrenna, Fram and Hinlopen-Yermak slides (green areas from south to north) (after [17,18]). (b) Local bathymetry with contour lines (white lines in meters), available 2D seismic profiles, location of the pipe structure (red dot) next to the Hinlopen-Yermak Slide (green area) and the location of the seismic profile shown in Fig. 2 (blue bold line).

Supplementary Table 1 | Pressure ratio of the pore pressure and overpressure at 215 mbsf.

	min	max
rho bulk [kg m^{-3}]	1690	2140
lithostatic pressure at 215 mbsf [kPa]	3564	4514
pore pressure* [kPa]	2614	2614
pressure ratio pore per lithostatic [%]	**73**	**58**

* hydrostatic plus overpressure

The pore pressure is defined as the sum of hydrostatic pressure at 215 mbsf and overpressure generated by 45 m gas column height. The lithostatic pressure is calculated for a minimal and maximal bulk density [15].

Supplementary Table 2 | Values for the parameters that are kept constant in the parametric studies in 45 and 215 mbsf.

depth [mbsf]	rho bulk [kg m^{-3}]	Poisson ratio	friction angle [°]	cohesion [kPa]	Biot-Willi const.
45	1700	0.3	25	0	0.72
215	1800	0.3	30	0.28	0.72

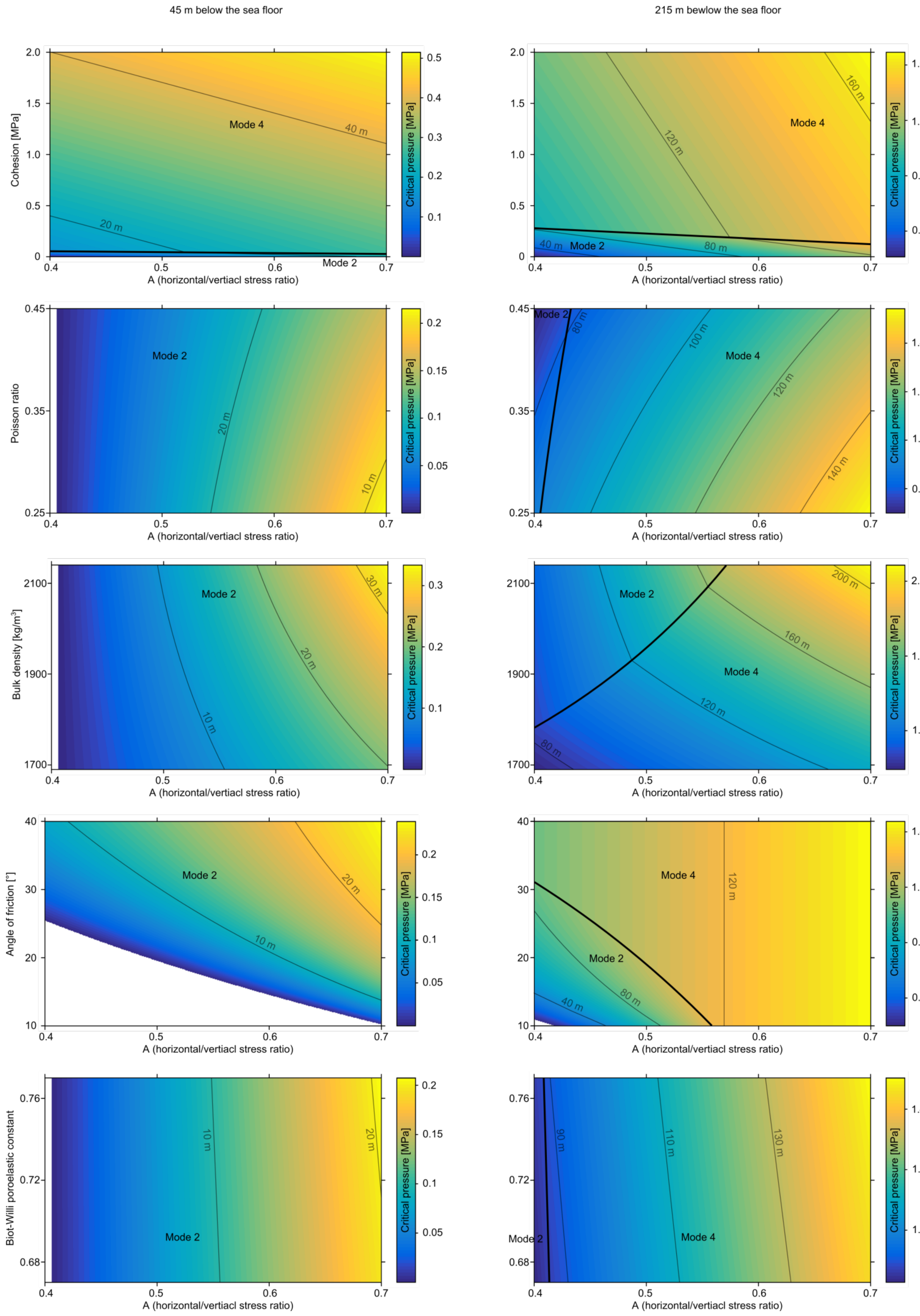
45 m below the sea floor
215 m bewlow the sea floor
Cohesion [MPa]
Mode 4
40 m
20 m
Mode 2
120 m
160 m
80 m
40 m
Critical pressure [MPa]
A (horizontal/vertiacl stress ratio)
Poisson ratio
20 m
10 m
100 m
140 m
Bulk density [kg/m³]
30 m
200 m
Angle of friction [°]
Biot-Willi poroelastic constant
90 m
110 m
130 m

Supplementary Figure 2 | Illustrations of the critical pressure (color code) for failure as a function of stress ratio and cohesion, Poisson ratio, bulk density, angle of friction and Biot-Willi poroelastic constant. Mode 2 and 4 refer to shear failure and tensile under compression, respectively [19]. The grey lines correspond to the critical gas column height, supposing 100% replacement of water by gas in 40 mbsf (left) and 190 mbsf (right).

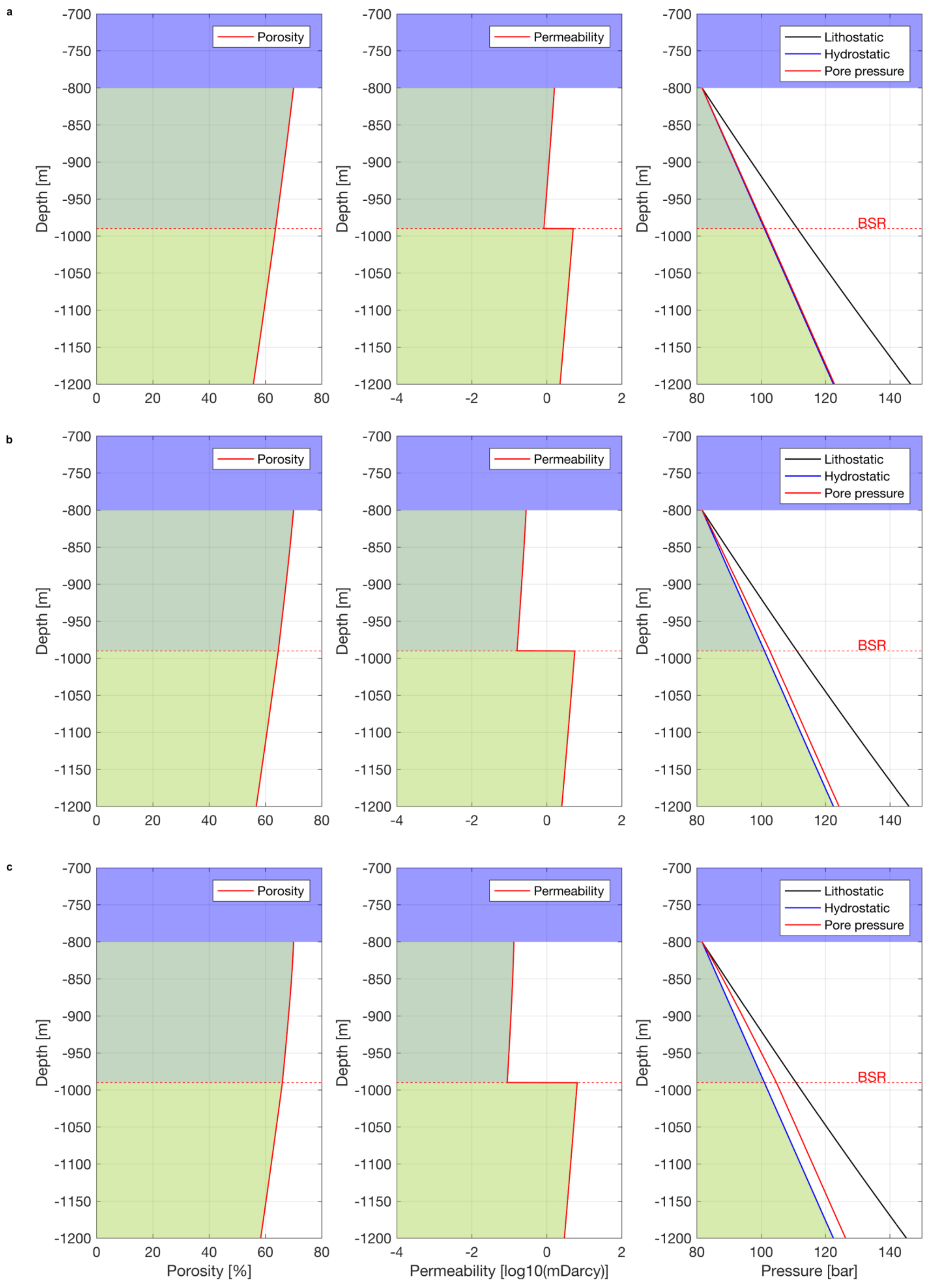
a
b
c
Porosity
Permeability
Lithostatic
Hydrostatic
Pore pressure
BSR
Depth [m]
-700
-750
-800
-850
-900
-950
-1000
-1050
-1100
-1150
-1200
0
20
40
60
80
-4
-2
0
2
80
100
120
140
Porosity [%]
Permeability [log10(mDarcy)]
Pressure [bar]

Supplementary Figure 3 | Calculated porosity, permeability and pressure depth-profile of sediments at 800 m water depth assuming different gas hydrate saturations above the BSR in the gas hydrate stability zone. The bottom of the blue area is the sea floor, the green area the subsurface, with 20 % (a), 50 % (b) and 60 % (c) gas hydrate saturation in the gas hydrate stability zone (dark green area). The presence of gas hydrates reduces the permeability and changes pore pressure during mechanical compaction. The hydrostatic pressure is elevated by 30 kPa (a), 170 kPa (b) and 380 kPa (c) overpressure.

Supplementary References

1. Dobson, M. R., O`Leary, D. & Veart, M. Sediment delivery to the Gulf of Alaska: source mechanisms along a glaciated transform margin. *Geological Society, London. Special Publications* **129,** 43-66 (1998).

2. Scholz, N. A., Riedel, M., Urlaub, M., Spence, G. D. & Hyndman, R. D. Submarine landslides offshore Vancouver Island along the northern Cascadia margin, British Columbia: why preconditioning is likely required to trigger slope failure. *Geo-Marine Letters* **36,** 323-337 (2016).

3. Paull, C. K., Buelow, W. J., Ussler, W. & Borowski, W. S. Increased continental-margin slumping frequency during sea-level lowstands above gas hydrate-bearing sediments. *Geology* **24,** 143-146 (1996).

4. Leslie, S. C. & Mann, P. Giant submarine landslides on the Colombian margin and tsunami risk in the Caribean Sea. *Earth and Planetary Science Letters* **449,** 382-394 (2016).

5. Krastel, S., et al. Sediment dynamics and geohazards off Uruguay and the de la Plata River region (northern Argentina and Uruguay). *Geo-Marine Letters* **31,** 271-283 (2011).

6. Ben-Avraham, Z., Smith, G., Reshef, M. & Jungslager, E. Gas hydrate and mud volcanoes on the southwest African continental margin off South Africa. *Geology* **30,** 927-930 (2002).

7. Sultan, N., et al. Dynamics of fault-fluid-hydrate system around a shale-cored anticline in deepwater Nigeria. *Journal of Geophysical Research* **116** (2011).

8. Li, A., Davies, J. R., Yang, J. Gas trapped below hydrate as a primer for submarine slope failures. *Marine Geology* **380,** 264-271 (2016).

9. Bugge, T., Belderson, R. & Kenyon, N. H. The Storegga Slide. *Philosophical Transactions of the Royal Society of London* **325,** 357–388 (1988).

10. Elger, J., et al. Chronology of the Fram Slide Complex offshore NW Svalbard and its implications for local and regional slope stability. *Marine Geology* **393**, 141-155 (2017).

11. Dewangan, P., et al. Seabed morphology and gas venting features in the continental slope region of Krishna-Godavari basin, Bay of Bengal: Implications in gas-hydrate exploration. *Marine and Petroleum Geology* **27,** 1628-1641 (2010).

12. Horozal, S., et al. Mapping gas hydrate and fluid flow indicators and modeling gas hydrate stability zone (GHSZ) in the Ulleung Basin, East (Japan) Sea: Potential linage between the occurrence of mass failures and gas hydrate dissociation. *Marine and Petroleum Geology* **80,** 171-191 (2017).

13. Bangs, N. L., Hornbach, M. J., Moore, G. F., Park, J. O. Massive methane release triggered by seafloor erosion offshore southwestern Japan. *Geology* **38,** 1019-1022 (2010).

14. Mountjoy, J. J., et al. Shallow methane hydrate system controls ongoing, downslope sediment transport in a low-velocity active submarine landslide complex, Hikurangi Margin, New Zealand. *Geochemistry, Geophysics, Geosystems* **15,** 4137-4156 (2014).

15. Shipboard scientific Party. Proceedings of the Ocean Drilling Program, Initial report, Volume 151. College Station, TX, 271 –318 (1995).

16. Ingólfsson, Ó. & Landvik, J. Y. The Svalbard–Barents Sea ice-sheet – Historical, current and future perspectives. *Quaternary Science Reviews* **64,** 33–60 (2013).

17. Vanneste, M., Mienert, J., Bünz, S. & Bunz, S. The Hinlopen Slide: A giant, submarine slope failure on the northern Svalbard margin, Arctic Ocean. *Earth and Planetary Science Letters* **245,** 373 – 388 (2006).

18. Haflidason, H., de Alvaro, M. M., Nygard, A., Sejrup, H. P. & Laberg, J. S. Holocene sedimentary processes in the Andøya Canyon system, north Norway. *Marine Geology* **246,** 86 – 104 (2007).

19. Rozhko, A. Y., Podladchikov, Y. Y. & Renard, F. Failure patterns caused by localized rise in pore-fluid overpressure and effective strength of rocks. *Geophysical Research Letters* **34,** L22304 (2007).
